# Diversity of Arbuscular Mycorrhizal Fungi in Rhizosphere Soil of Maize in Northern Xinjiang, China, and Evaluation of Inoculation Benefits of Three Strains

**DOI:** 10.3390/jof12010027

**Published:** 2025-12-29

**Authors:** Ziwen Zhao, Wenqian Zhang, Wendan Xie, Yonghui Lei, Yang Li, Yanfei Sun

**Affiliations:** 1 Xinjiang Production and Construction Corps Key Laboratory of Oasis Town and Mountain-Basin System Ecology, Ministry of Education Key Laboratory of Xinjiang Phytomedicine Resource Utilization, College of Life Sciences, Shihezi University, Shihezi 832000, China; 20232006046@stu.shzu.edu.cn (Z.Z.); zhangwenqian0124@163.com (W.Z.); 20232006075@stu.shzu.edu.cn (W.X.); 2 Department of Plant Protection, College of Agriculture, Shihezi University, Shihezi 832000, China; lyh_agr@shzu.edu.cn

**Keywords:** AMF, maize, rhizosphere, diversity, soil factor, growth promoting effect

## Abstract

Arbuscular mycorrhizal fungi (AMF), which significantly enhances the absorption capacity of plant roots, forms a mutually beneficial symbiotic relationship with plants and is known as the “underground internet of plants”. To explore the community characteristics, environmental driving factors, and growth-promoting effects of AMF on maize in saline–alkaline habitats, this research attempts a survey of the rhizosphere soil of saline–alkali maize fields in four areas of northern Xinjiang (20 samples). High-throughput sequencing and morphological methods were used to analyze the diversity of AMF, and the correlation analyses of Mantel and Pearson were used to explore the relationship between AMF and soil environmental factors. The results showed that eleven genera of AMF belonging to three orders and seven families were identified in the rhizosphere soil of maize in Xinjiang, and *Glomus* was the absolute dominant group. The relationship analysis of the environmental factors and diversity of AMF shows that total nitrogen, total potassium and acid phosphatase are the main factors affecting the community structure of AMF. Through spore isolation and pot experiments, *Rhizophagus intraradices*, *Acaulospora denticulata* and *Glomus melanosporum* were successfully screened and identified. Among them, *Rhizophagus intraradices*, which can effectively improve the plant biomass, promote the root growth and enhance the absorption of phosphorus and potassium nutrients, promoted the growth of maize remarkably. This study systematically revealed the diversity of AMF as an environmental driving mechanism as well as plant growth promoter, establishing it as a candidate for application in the maize rhizosphere in northern Xinjiang. This provides a theoretical basis for AMF resource development and agricultural application in this saline–alkali area.

## 1. Introduction

Arbuscular mycorrhizal fungi (AMF) belong to a class of obligate living commensal microorganisms which can form a longstanding mutualistic symbiotic relationship with more than 80% of terrestrial plants on Earth [1]. The core feature of this symbiotic system is that AMF hyphae invade the cells of plant root cortices to form arbuscules structures, which serve as the key interface for nutrient exchange between host and fungus. At the same time, its extensive exophytic hyphal network significantly expands the absorption range of plant roots [2]. Studies have shown that the composition and distribution of AMF communities are significantly affected by geographic environment, vegetation type and soil factors [3,4]. For example, studies in the Taibai Mountain region (660–3500 m above sea level) in the Qinling Mountains have shown that AMF richness has a unimodal distribution along the coastal uplift gradient, with the highest diversity occurring in the mid-and low-altitude (approximately 1400 m) regions, with *Glomus* as the dominant genus [5]. The community structure and diversity of AMF are shaped by complex interactions between environmental and biological factors. Soil properties (such as pH, organic matter content and nutrient availability), land use patterns and host plant characteristics all significantly affect AMF colonization and community construction. There are significant differences in AMF community composition within different ecosystems: for example, genera such as *Scutellospora* are dominant in tropical rain forests, while *Diversispora* and *Scutellospora* [6] are common in temperate forests, and *Glomus* is dominant in agricultural systems and desert ecosystems. Plant functional groups have also played a role in regulating AMF composition; for example, legumes could increase AMF abundance, while non-mycorrhizal plants (such as *Carex flacca*) might indirectly affect AMF diversity by changing the mycorrhizal infection pattern [7,8].

In agro-ecological systems, the regulating effects of the soil’s physical and chemical properties on AMF diversity have been particularly prominent [9]. Long-term nitrogen addition (>6 years) reduced AMF species richness and hyphal network stability, and indirectly weakened AMF ecological function by reducing plant diversity [6,10]. Excessive fertilizer application generally inhibited the colonization of AMF, while crop rotation and organic additives contributed to its diversity [11]. As AMF is highly sensitive to environmental changes, its diversity, community structure and ecological function are vulnerable to a variety of factors, such as plant type and the soil’s physical and chemical properties [12]. These soil properties affect microbial community composition and soil health by altering the rhizosphere microenvironment and ultimately have a profound impact on ecosystem productivity [13]. Functionally, AMF are able to significantly enhance the absorption efficiency of host plants as to mineral elements such as phosphorus, nitrogen and zinc, especially phosphorus with poor mobility, and improve the drought resistance, disease resistance and resistance to heavy-metal stress, as well as promote the formation of soil aggregates and improve the soil structure [14]. Therefore, analyzing the biogeographic distribution pattern of AMF and its driving mechanisms, especially the roles of key soil factors, is of great significance for ecosystem protection and the construction of an agricultural microbial germplasm resources bank [15,16]. In this context, AMF is considered to be a key biological factor driving sustainable agricultural development and reducing fertilizer and pesticide inputs [17].

Conventional AMF identification relies on spore morphology, including size, wall layer and surface texture [18]. However, under different environmental conditions, morphological characteristics may vary greatly, resulting in difficulties in accurate identification. In addition, some AMF species produce few or no spores under field conditions [19]. These limitations have driven the widespread adoption of molecular technology since the 1990s. High-throughput sequencing (HTS), in particular, the Illumina NovaSeq-based amplicon sequencing, has become a powerful tool for delineating the composition and diversity of AMF communities without incubation [20]. Studies using high-throughput sequencing technology have shown that the abundance and diversity of AMFs are severely underestimated in many environments [21,22,23]. Target regions such as Small Subunit (e.g., primers AMV4.5NF/AMDGR) have been widely used for AMF molecular analysis, providing higher resolution and more accurate species-level identification compared with earlier methods such as RFLP or DGGE, significantly increasing the depth and breadth of the recognition of AMF community structures [24,25,26,27,28,29].

As one of the most important food and feed crops in China and the world, maize plays an irreplaceable role in ensuring national food security, promoting agricultural economic development and maintaining social stability, and its high and stable yield is extremely important in terms of strategic significance [30]. As it is a typical AMF symbiotic dependent crop, the growth and yield formation of maize are closely related to the structure and function of the AMF community in the rhizosphere [31]. Xinjiang, as an important maize-producing area in China, has a unique and fragile agro-ecological environment. The soil in this area is generally limited by high pH, low organic matter content, limited available phosphorus fixation and low rainfall [32]. These adverse soil conditions are theoretically more conducive to AMF playing its ecological function, because mycorrhizal symbiosis is an important strategy for plants, used to adapt to nutrient stress environments [33]. AMF is widely believed to enhance plant resistance to abiotic stresses such as drought and salinity. Under drought conditions, AMF colonization improved plant water acquisition and reduced oxidative stress through enhanced antioxidant activity and osmoregulation [34]. Similarly, in saline soils, AMF reduced ion toxicity and oxidative damage while increasing nutrient uptake and photosynthetic efficiency [35]. Studies have shown that AMF inoculation has been shown to improve the stress resistance of many crops, including maize [36,37]. However, modern intensive agricultural practices (such as high-intensity chemical fertilizer application and continuous cropping) will significantly inhibit the diversity and function of the soil’s AMF community [38,39].

In recent years, although there have been studies on AMF communities in some ecological regions in Xinjiang (such as Tianshan Mountain, Yili Valley and Junggar Basin) [40,41,42], and there have also been reports on the AMF diversity of crops such as cotton and *Ferula Sinkiangensis* [23,40]. There is still an obvious research gap in maize rhizosphere AMF in the saline–alkali areas of northern Xinjiang: there is a lack of systematic investigation of the structure and spatial distribution characteristics of AMF communities in the main maize-producing areas in this region, and the existing studies have not focused on maize. In addition, the key driving forces associated with the physical and chemical factors such as high EC and low available phosphorus that are unique to saline–alkali soil in northern Xinjiang with respect to AMF community differentiation are not clear, so it is difficult to support the development of targeted microbial resources. At the same time, there is a lack of isolation, purification and functional verification of locally dominant AMF strains, which limits the research and development and application of regional specific mycorrhizal biofertilizers. Using the research system of “from community to function” (combined with high-throughput sequencing, morphological identification and pot test) to analyze these scientific problems will not only help to develop the mycorrhizal biofertilizer (which refers to the microbial preparation made by standardized propagation with AMF strain as the core) of AMF germplasm [15], but also clarify the important role of symbiotic relationship in the protection of germplasm resources and provide strategic support for agricultural green transformation [43].

Therefore, the purposes of this study were as follows: (1) to study the structure and diversity of the AMF community in the maize rhizosphere in different ecological regions of northern Xinjiang using high-throughput sequencing technology; (2) to clarify the key soil environmental factors driving the differentiation of AMF communities in this saline–alkaline habitat; and (3) to isolate and propagate dominant AMF strains from the maize rhizosphere, and evaluate their growth-promoting effects on maize seedlings under saline–alkaline stress so as to identify high-efficiency functional strains for agricultural application. The research results are expected to provide theoretical bases and microbial resources for improving crop resistance and promoting the sustainable development of agriculture in arid areas.

## 2. Materials and Methods

### 2.1. Sample Collection

Samples were collected from spring-sown maize fields in northern China. The selected plots are all representative saline–alkali farmlands with long-term (≥5 years), continuous maize cultivation. The sampling locations are in Table S1. The tested variety was Xinyu 41, a salt-tolerant hybrid, which was planted from late April to early May, and the maize was in the filling stage (R5 stage) at the time of sampling (mid-August).

Soil samples were collected at selected maize root areas. Samples were collected at each sampling point using the five-point sampling method. After the topsoil, stones and debris were removed, the rhizosphere soil (approximately 10–30 cm deep) and the root system were collected near the roots with a sterile tool. The collected samples were stored in a self-sealing bag, labeled, placed in an ice box, and taken back to the laboratory for storage in the −80 °C ultra-low temperature refrigerator.

Soil samples were divided into two parts; one part was placed into a sterilized 50 mL centrifuge tube (soil and root) for fungal 18S rDNA high-throughput sequencing. The other part of the soil sample was naturally air-dried and sieved through a 2 mm separating sieve for soil physicochemical determination. After the roots were cleaned, they were immersed in FAA fixative and stored in a 4 °C refrigerator for root infection experiments.

### 2.2. Soil DNA Extraction and High-Throughput Sequencing

Using the Illumina NovaSeq high-throughput sequencing technology of Beijing, China Biological Engineering Co., Ltd., it was determined that the paired-end length was 380 bp. The sequencing depth was designed in the context of an average of ≧60,000 original sequencing sequences per sample. The primer was AMV 4.5 NF (5′-AAGCTCGTAGTTGAATTTCG-3′); AMD GR (5′-CCCAACCATTCCAATCAT-3′) [44], and ASV clustering analysis, species annotation and database comparison were conducted. The original image data file obtained by high-throughput sequencing (using a sequencing platform such as Illumina NovaSeq) was converted into the original sequencing sequences (sequenced reads) after base recognition (Base Callin8 g) analysis. The results were stored using a file format FASTQ (fq.) that contains sequence information of the sequencing sequences (Reads) and the corresponding sequencing quality information. Three suitable repeat units were set for each sample.

In data pretreatment, taking a “quality first” approach, raw reads obtained by sequencing were filtered using Trimmomatic v0.33 software. Then, the primer sequences were identified and removed using cutadapt 1.9.1 software to obtain clean reads that did not contain primer sequences. DADA2 denoising using QIIME2 2020.6 [45] Middle dada2 [46] Methods Denoising, double-ended sequence concatenation and removal of the chimeric sequence were performed to obtain the final non-chimeric reads. Clean reads then were conducted on feature classification to output the ASVs (amplicon sequence variants) by dada2 [46], and ASVs counts less than 2 in all samples were filtered. Taxonomy annotation was performed, based on the Naive Bayes classifier in QIIME2 [47] using the SILVA database [48] (release 138.1), with a confidence threshold of 70%.

In addition, the AMV4.5NF/AMDGR primer pair used in this study is mainly aimed at the SSU region of AMF 18S rDNA. The regional variation is relatively conservative, which is suitable for reliable community structure analysis and diversity assessment at the genus level. However, its resolution is usually not enough to achieve accurate species-level identification. Therefore, based on the community composition analysis of the high-throughput sequencing and the subsequent correlation analysis with environmental factors, the work of this paper has been mainly functioning at the genus level. Accurate identification of species level is achieved by combining the morphological identification (spore morphology) described in Section 2.3 and Section 2.5 with multi-segment sequencing of the pure cultured strains.

### 2.3. Root Colonization and Spore Isolation and Identification of AMF

In determining the infestation, the maize roots, the infection rate of which was determined to correspond to the soil samples, were collected from 20 plots. After washing with clear water to remove soil impurities, they were stored in a refrigerator at 4 °C with the FAA fixed solution. Staining [49] was performed using the method described by Phillips and Hayman (1970), and the cross method was used [50]. The structures of plexuses, hyphae and vesicles after AMF infection were observed under the light microscope Olympus CX21 (Olympus Corporation; Tokyo, Japan). Calculation of root infection rate was as follows:

$$Infectionrate\;(100\%)=\frac{Total\;crossing\;points-Sum\;of\;noninfectious\;points}{Total\;crossing\;points}\times 100\%$$

AMF spores [51] were isolated by wet-sieving sucrose gradient centrifugation. The morphological characteristics of spores were observed under a microscope. The morphological identification of the AMF was mainly based on the AMF classification website and books. The pictures and descriptions of species and genera provided by the AMF professional classification website (http://www.amf-phylogeny.com, accessed on 18 December 2024), the International AMF Collection INVAM (http://invam.wvu.edu/home, accessed on 20 December 2024), the Polish Agricultural University (http://www.zor.zut.edu.pl/, accessed on 20 December 2024) and Myco Bank Database (http://mycobank.org/, accessed on 24 December 2024) were also included, and morphological identification was conducted with reference to other literature-based classification materials [52,53]. The calculated ecological parameters included spore density, species richness, isolation frequency, relative abundance and important values (Table S2).

### 2.4. Determination of the Soil’s Physical and Chemical Properties and Enzyme Activity

Soil physicochemical properties [23] and soil enzyme activity [54] tests were performed on the treated soil samples. The method and place of sample collection were consistent with the procedures described in Section 2.1. (The preparation method was as follows: samples were naturally air-dried in a ventilated and dust-free environment, gravel was removed, and plant residues were processed through a 2 mm sieve and further ground through a 0.15 mm sieve for the determination of the soil’s physical and chemical properties and enzyme activity. The measurements were in accordance with the national standards for research in China: soil pH (potentiometric) [55], conductivity, organic matter (potassium dichromate–sulfuric acid oxidation) [56], total nitrogen/available nitrogen (Kjeldahl method and alkali diffusion method) [57,58], phosphorus (acid digestion and bicarbonate extraction) [59], potassium (flame photometry and ammonium acetate extraction) [55], and enzyme activities (sucrose-3,5-dinitrosalicylic acid colorimetric method, protease-ninhydrin method, and acid phosphatase-Solabio test kit) [60].

### 2.5. Single Spore Propagation and Identification

After surface disinfection, maize seeds (Xinyu 41) were sown in sterilized matrix (peat: vermiculite: perlite = 1:1:1), and when the seedlings grew to a certain stage, they were used to inoculate AMF spores. AMF spores were separated by wet sieve decantation-sucrose centrifugation and then sterilized to carry out single spore propagation. The sterilize substrate was filled to about 2/3 of the height of a flowerpot, and clean spores were drawn under a stereomicroscope, placed on the primary root or lateral roots of maize seedlings, and immediately put into the flowerpot. The seedlings were then righted and filled with the substrate [61,62]. The small subunit genes of spore (primers AML1/AML2 [63] and AMV4.5NF/AMDGR [44]) DNA were amplified by PCR, and the PCR products were detected by agarose gel electrophoresis and sent to Beijing, China Biological Engineering Co., Ltd. for sequencing and sequence alignment.

**Figure 1:**
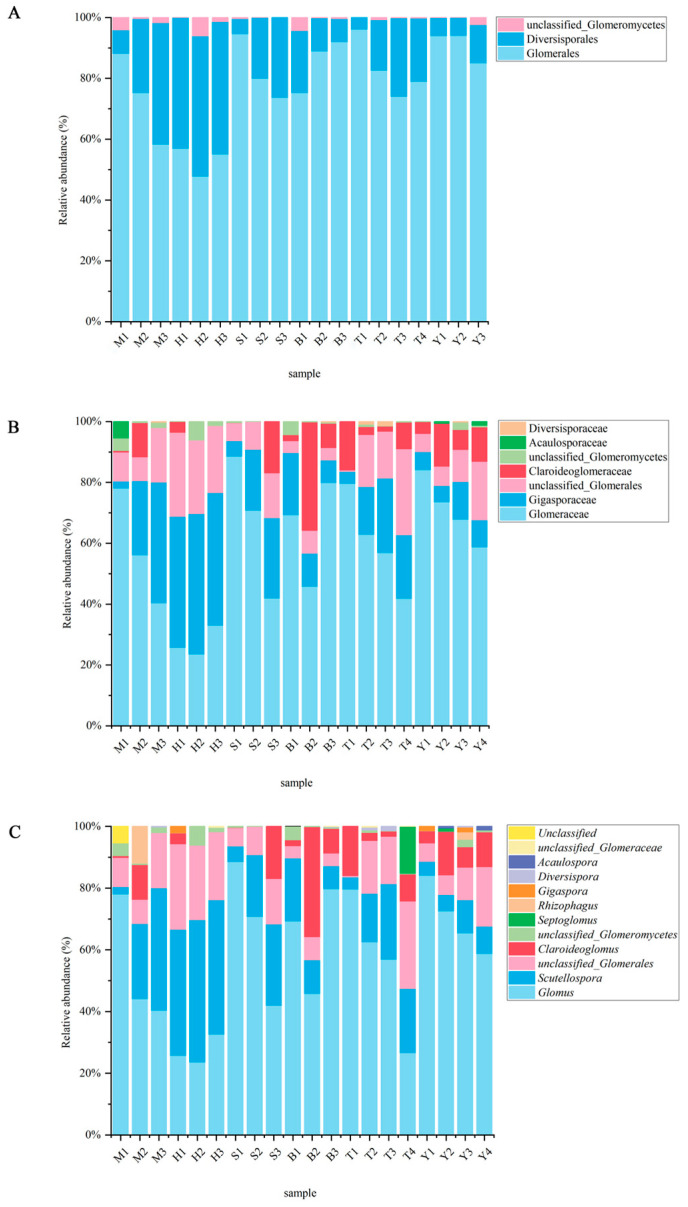
Relative abundance distribution of AMF at different levels in maize rhizosphere soil in northern Xinjiang: (**A**) order level; (**B**) family level; and (**C**) genus level. Each column represents a sample, and the height of the column indicates the relative abundance of the corresponding taxon.

Based on 18S rDNA sequence, multiple sequence alignment (Clustal W) was performed with MEGA 11. Archaeospora leptoticha was taken as the outer group, the Kimura 2-parameter model and adjacency method (NJ) were used to build trees, and a Bootstrap test was conducted for 1000 repetitions (see Figure 1 for parameter settings). The target sequence was compared with the NCBI model strain sequence to determine the taxonomic status of the strain.

### 2.6. Potted Maize Growth-Promoting Experiment

Inoculation treatments included single and compounded AMF strains. The substrate used in the pot experiment was the same as that in Section 2.5. The AMF strains used in experiments were all derived from pure culture strains which had been successfully propagated by the single spore in the work described in Section 2.5. The inoculation amount of each pot was 250–300 spores [64]; the inoculation amount of each pot of compound strain treatment (*RA*, *RG*, *AG*, *RAG*) was equal to the proportion of each single bacterial agent, and the total spore number was consistent with that of the single-strain treatment. The inoculation method used was to spread the microbial inoculum evenly around the root system of the maize seedlings [65]. The blank control CK (plants that had not been inoculated with the target AMF live bacteria) was set. All other culture conditions were completely consistent with inoculation treatment, with 3 biological replicates utilized for each treatment. The growth effect was evaluated after 30 days. Relevant root system parameters such as plant height, fresh/dry weight, root length, surface area and volume were analyzed using an Expression 1100XL (Epson; Beijing, China) scanner after harvesting and drying. Determination of chlorophyll content was performed by ethanol extraction [66]. The root activity was determined by the TTC method [67]. The contents of major elements (nitrogen, phosphorus and potassium) in the samples digested with catalyst and concentrated sulfuric acid were determined by the Kjeldahl method [68], molybdenum–antimony colorimetric method [69] and flame photometric method, respectively [70].

### 2.7. Data Processing

The data were processed in Microsoft Excel, and SPSS 26 was used for one-way analysis of variance, descriptive statistics, correlation analysis and principal component analysis. Photoshop 2025 and GraphPad Prism 9.5.1 were used for image rendering.

## 3. Results

### 3.1. AMF Community Composition and Diversity

In this study, the high-throughput sequencing results associated with the 20 samples collected from maize rhizosphere soil in the four regions were analyzed, and 2081 amplicon sequence variants (As Vs) were identified. The sample dilution curves all reached the plateau (Figure S1), indicating that the current sequencing depth was sufficient. According to taxonomic notes, the AMF soil communities in the maize rhizosphere were identified as eleven genera, seven families and three orders (Table 1). The order Agglomerates was the absolutely dominant group, with the average relative abundance reaching 79.01%. In the classification of families, Conglomerate was dominant (58.94%). The horizontal composition analysis of genus showed that an average relative abundance of 57.37% was sufficient to constitute the dominant genus in *Glomus*. In contrast, the genera *Diversionary* (0.15%), *Scutellospora* (0.09%) and unclassified_*Conglomerate* (0.04%) were all rare (Figure 1) (relative abundance < 1%). In addition, among the 11 genera identified in this study, the cumulative relative abundance of unclassified taxa (unclassified_*Glomeraceae*, unclassified_*Glomerales*, unclassified_*glomerates*) was 3.6%. The existence of this classification is mainly related to the insufficient resolution of SSU rDNA primers and the lack of reference sequences for some rare taxa, which are all low-abundance taxa, and would not have had a substantial impact on the community structure analysis of the core dominant genera (*Glomus*, *Rhizophagus*, *Acaulospora*, etc.).

**Figure 2:**
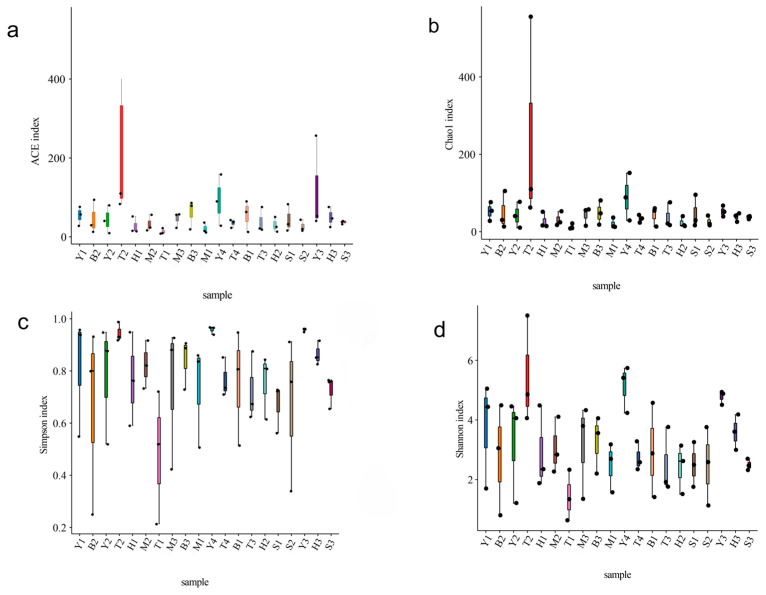
Alpha diversity index of the AMF community in maize rhizosphere soil in northern Xinjiang: respectively, the (**a**) ACE index, (**b**) Chao1 index, (**c**) Simpson index, and (**d**) Shannon index. Markers with different colors represent multi-sample or grouped information, and each marker point extends with vertical lines, showing the dispersion degree and numerical range of the data under various categories.

Alpha diversity analysis revealed that there were significant differences among plots in the rhizosphere soil community of maize (Figure 2, Table S3). Among them, the ACE index (249.84 ± 152.76), Chao1 index (242.13 ± 157.04) and Shannon index (5.47 ± 1.04) of the T2 plot were significantly higher than those of other plots (*p* < 0.05), indicating that it had the highest species richness, diversity and community uniformity. The values for the Simpson index (0.96 ± 0.00) and Shannon index (5.13 ± 0.46) associated with the Y3 and Y4 plots are at a high level, indicating that the community uniformity is good. On the contrary, the T1 plot has the lowest value among all indices (ACE: 13.91 ± 4.41, Chao1: 12.67 ± 4.18, Shannon: 1.43 ± 0.49), while the S1 plot has the lowest ACE index (21.58 ± 7.85), reflecting the decrease in AMF community richness and diversity. These results show that the ecological environment and soil conditions of the sample plot may have an important impact on the overall richness and diversity of the AMF community.

**Figure 3:**
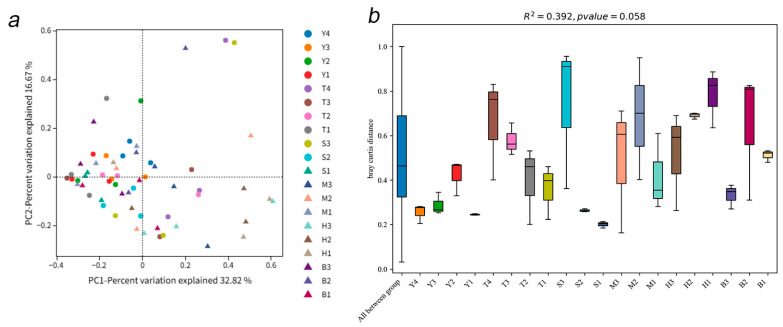
PCoA (**a**) and PERMANOVA (**b**) analyses of the AMF community in maize rhizosphere soil in northern Xinjiang. (**a**) Each point represents a sample, and points with different colors and patterns represent different groups. PC1 represents the first principal component of AMF, and PC2 represents the second principal component. PC1 and PC2 can usually explain the largest proportion of variation in the data set. (**b**) The distance and test between samples are represented by the block diagram and line diagram, the abscissa represents the distance between all samples, and R^2^ represents the degree to which the grouping method explains the sample differences.

**Figure 4:**
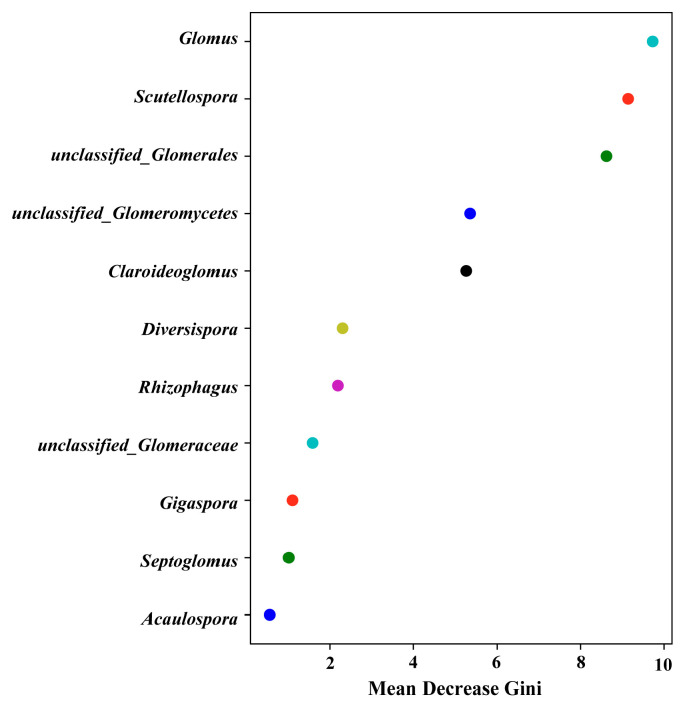
Random forest analysis based on the differences in AMF community structure in maize rhizosphere soil (genus level). Each point in the figure represents a specific genus of AMF, and its position on the horizontal axis reflects the average reduction value of the genus. For example, the closer the point is to the right side of the horizontal axis, the greater the corresponding average reduction, that is, the fungus genus is more important in correlation analysis or model construction.

PC1 explained 32.82% of the community variation, PC2 explained 16.67%, and the first two axes explained about 49.49% of the variation, indicating that it can effectively capture the core differentiation trend of the community. The community structures of B1, B2 and B3 showed obvious aggregation (Figure 3a). PERMANOVA analysis showed that *R*^2^ = 0.392 (grouping explained about 39.2% of the community differences) and *p* = 0.058 (close to the significance level of 0.05), indicating that the influence of grouping on the community differences was close to statistical significance (Figure 3b). The surplus has a certain screening effect on the community structure of AMF genus level, but the unexplained 60.8% variation needs to be explained by other factors (such as soil physical and chemical factors and microclimate). The communities in groups S3 and B1 are quite different from those of the other plots. However, the Y3 and Y2 communities have greater degrees of similarity relative to the other plots. The results show that there are significant differences in the AMF community structure in different places, and the associated change pattern may be caused by the nonlinear interaction of the soil’s physical and chemical properties. Simple grouping factors provide limited explanation for the community structures. In random forest analysis (Figure 4), the Mean Decrease Gini index of *Glomus* was found to be higher than those of other taxa, indicating that the difference in the AMF communities between different plots is mainly reflected in the dominant genus.

### 3.2. Relationship Between AMF Diversity and Soil Properties

**Figure 5:**
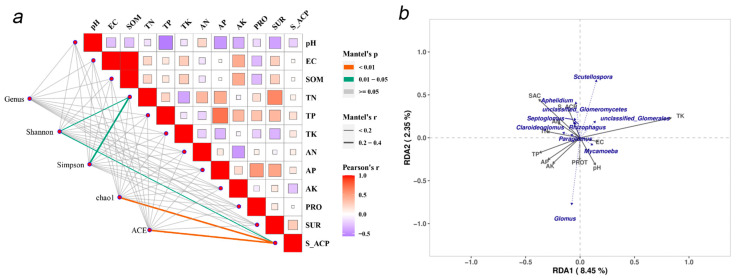
(**a**) Visual map of correlations between AMF community (genus level) diversity index and the soil’s physical and chemical properties in maize rhizosphere soil, based on the Mantel test and Pearson correlation analysis. TK: Total potassium; TN: Total nitrogen; TP: Total phosphorus; AK: Available potassium; AN: Available nitrogen; AP: Available phosphorus; SOM: Soil organic matter; EC: Electrical conductivity; PRO: Protein; SUR: Sucrase; S_ACP: Soil acid phosphatase. The Mantel test is used to evaluate the overall correlation between AMF community structure at the genus level and soil’s physical and chemical properties. Different colors in the legend represent the range of the Mantel test correlation coefficient, such as orange 0–0.1 and green 0.01–0.05. Pearson correlation analysis: the Pearson correlation coefficients between the indicators of the soil’s physical and chemical properties and between indicators and the AMF community diversity index are displayed using color depth (white to red). The deeper the red is, the stronger the positive correlation is, while the light or white color is weak, and the negative correlation may be in reverse color gradient. (**b**) Redundant analysis (RDA) bisequence diagram of the generic AMF community (dotted line) and the soil’s physical and chemical factors (full line). The length of the arrow reflects the influence intensity of the factors on community variation, and the direction of the arrow represents the action direction of the factors. The text label represents AMF genus.

The determination and analysis of the soil’s physical and chemical properties and enzyme activities for the 20 sample plots (Tables S4 and S5) showed that there existed significant differences in pH, EC, SOM, AP and AK among the different plots, showing the heterogeneity of soil across all the plots. The T2 sample plot with the highest species diversity had the highest TK content, while AP and TP were lower. The correlation network heat map was constructed based on the microbial diversity index and soil parameters (Figure 5a). It shows that in the rhizosphere soil, TN was significantly correlated with the Shannon and Simpson indices (0.01 < *p* < 0.05). S_ACP activity was significantly correlated with the Shannon, ACE and Chao1 indices (0.01 < *p* < 0.05 or *p* < 0.01); the higher the activity of S_ACP, the higher the species richness of AMF community. However, pH and EC were negatively correlated with diversity index. In RDA analysis (Figure 5b), the TK arrow points to the right of RDA1, which is highly consistent with the direction of Scutellospora, indicating that TK is positively correlated with the abundance of Scutellospora, which prefers high-potassium soil habitats. The arrow of S_ACP points to the left of RDA1, which is consistent with the direction of Septoglomus, indicating that the activity of S_ACP is positively correlated with the abundance of these genera, and the genus may participate in the soil phosphorus cycle (S_ACP improves available phosphorus). The results showed that TK, TN and S_ACP significantly affected Alpha diversity in rhizosphere soil. A high EC and high pH environment will inhibit the diversity of AMF groups.

**Figure 6:**
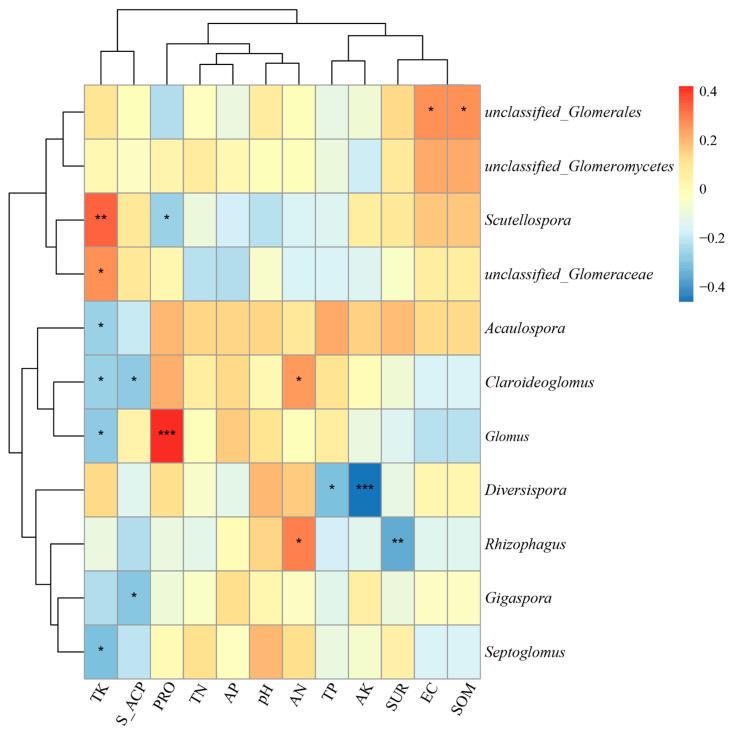
Heat map of correlation between AMF community in inter-root soil at the genus level and the soil’s physico-chemical properties and enzyme activities. The color gradient is from blue to red, representing a range of correlation coefficient from −0.4 to 0.4, with blue indicating negative correlation and red indicating positive correlation. “*” denotes *p* < 0.05, “**” denotes *p* < 0.01, “***” denotes *p* < 0.001; the more asterisks, the more significant the correlation.

An analysis based on the heat map of correlation between AMF communities at the genus level and soil factors (Figure 5) showed that *unclassified*_*Glomerales* had a significant positive correlation with EC and SOM, suggesting that they were enriched in the habitat with high salinity and rich organic matter. A significant positive correlation was observed between the genera *Claroideoglomus* and *Acaulospora* and AN, indicating that N availability drove their colonization. *Scutellospora* and *unclassified*_*Conglomerate* had highly significant positive correlations with TK, indicating a preference for potassium-rich environments, while *Scutellospora* and *Septoglomus* (r = −0.61, *p* = 0.015) tended to low-potassium habitats. *Glomus* had an extremely significant positive correlation with PRO, suggesting that it might play a role in nitrogen cycling (Figure 6). In summary, the correlations between most AMF genera and AP were weak or significantly negative, indicating that AP generally inhibited AMF groups. In particular, TK showed a significant correlation with most AMF genera, so TK was the primary driver of structural differentiation in AMF communities.

### 3.3. Isolation, Identification and Colonization of Maize Rhizosphere AMF

A total of 14 species (nine genera, six families, three orders) of AMF were isolated from the soil samples collected from 20 plots in the four regions (Table 2). As shown in Table 3, most AMF species were distributed unevenly, with significant differences in isolation frequency (IF), relative abundance (RA) and important value (IV) among different species. Among them, *Gloms melanoma* was the dominant species, with the IF, RA and IV reaching 95.00%, 38.33% and 66.66% respectively, showing extensive distribution and dominant position. In addition, some species were rare, such as *Diaspora legerdemain* (IF = 10.00%, RA = 3.38%, and IV = 6.69%) which only appeared in the H2 and T4 sample plots. The rare species had little ecological effect and showed significant spatial limitation.

The observation of stained root segments showed that AMF showed significant infection difference in different plots, with the total infection rate ranging from 56.79% to 83.51%, and an average rate of 70.64% (Table 4). The plots with high total infection rates were M2 (83.51%), S1 (79.38%) and T2 (78.31%). In the 20 plots, the infection rates of hypha, bush branch and vesicle were 15.38–47.51%, 3.85–34.12% and 0.31–37.18%, respectively. The infection rates of bush branch of B1 and vesicle of H2 were the lowest (3.85% and 0.31%); it was difficult to observe the corresponding structure. Among the four areas, Ilikazak Autonomous Prefecture has the highest AMF rating rate. On the whole, the hyphal infection rate was higher than that of vesicle and cluster branch, indicating that hyphal infection was the main form and determined the total infection rate to a large extent. The results show that there are great differences in infection rate of mycorrhizal fungi and the structural composition of AMF spores in the different regions, which may affect the plants’ dependence on arbuscular mycorrhizal fungi and their interaction with the soil.

Pearson correlation analysis (Table 5) of soil factors and the root infection rate showed that the absolute values of Pearson correlation coefficients of the soil factors and root infection rate were small, and the significance levels failed to reach the threshold (*p* > 0.05). There was no significant correlation between root infection rate and most soil factors, except for a weak negative correlation trend of organic matter (SOM) (r = −0.116), indicating that soil factors had no significant regulatory effect on infection rate.

### 3.4. Inoculating AMF to Promote the Growth of Maize Seedlings

**Figure 7:**
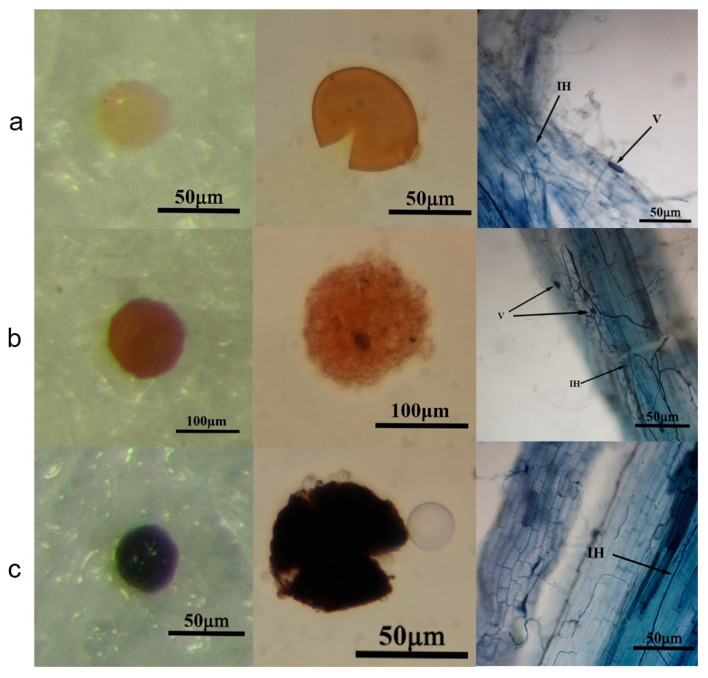
Morphology of three kinds of spores isolated and propagated from maize rhizosphere soil and their infected root system map: (**a**) *Rhizophagus intraradices* (*R.*), (**b**) *Acaulospora denticulata* (*A.*), (**c**) *Glomus melanosporum* (*G.*). From left to right: spore morphology map, spore staining map and root infection map. V (Vesicle) describes the vesicle, IH (Intraradicular Hyphae) describes the intra-root mycelium.

The root staining in 28 samples confirmed the existence of infectious structure. Sucrose-wet sieving showed significant spore proliferation in the three AMF species, with spore densities of 150–200/50 g, 200–300/50 g and 100–120/50 g matrices, respectively. Three megaspores, No.1 (*Rhizophagus intraradices*), No.2 (*Acaulospora denticulata*) and No.3 (*Glomus melanosporum*), were identified and designated (Figure 7). The phylogenetic tree is shown in Figure S2.

**Figure 8:**
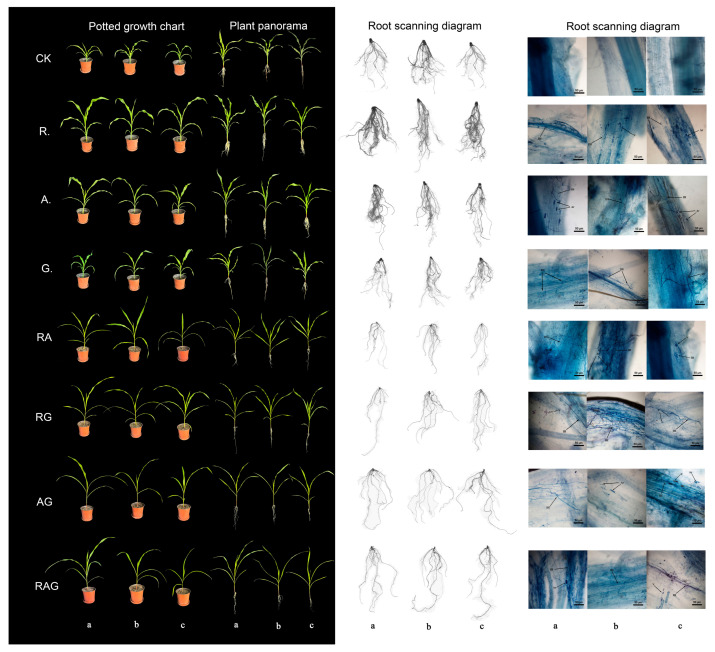
Potting, root promotion effect and root infestation map of different treatments on maize seedlings. Blank control CK refers to the target AMF that has not been inoculated. *R.*, *A.*, *G.*, *RA*, *RG*, *AG* and *RAG* are different treatments, that is, single microbial inoculum and compound microbial inoculum inoculation; a, b and c are three repetitions of unified treatment. In the root infection map, V (Vesicle) describes the vesicle, IH (intrinsic hyphae) describes the root hyphae and Ar (Arbuscules) describes the clump structure.

**Figure 9:**
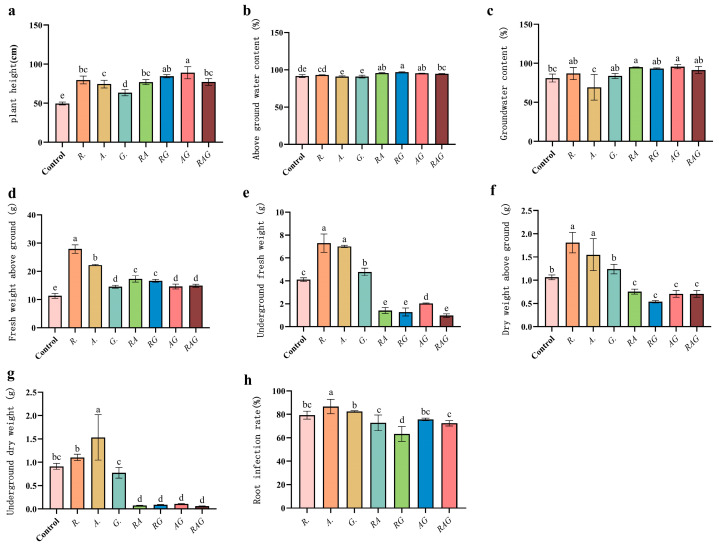
Effects of different treatments on the biomass of maize seedlings. Abscissa *R.*, *A.*, *G.*, *RA*, *RG*, *AG* and *RAG* are different treatments, that is, single microbial inoculum and compound microbial inoculum inoculation. (**a**) Plant height, (**b**) Above-ground water content, (**c**) Groundwater content, (**d**) Fresh weight above ground, (**e**) Underground fresh weight, (**f**) Dry weight above ground, (**g**) Underground dry weight, (**h**) Root infection rate. On the error lines a–e of each graph, it is to judge whether the difference between groups is significant (*p* < 0.05).

**Figure 10:**
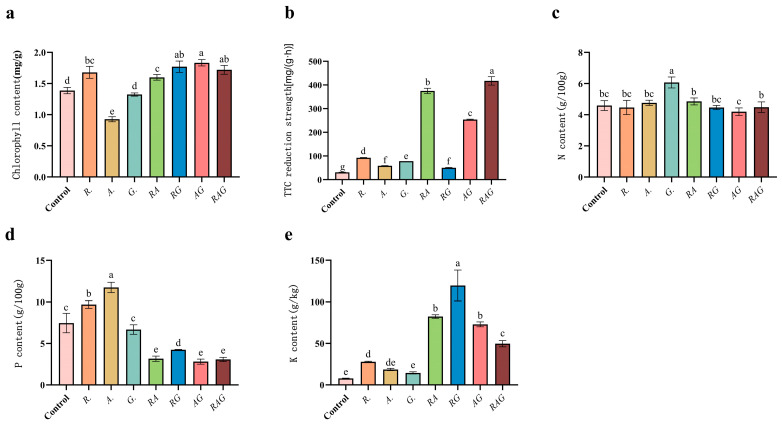
Effects of different treatments on the macroelement absorption, chlorophyll content and root activity of maize seedlings. Abscissa *R.*, *A.*, *G.*, *RA*, *RG*, *AG* and *RAG* are different treatments, that is, single microbial inoculum and compound microbial inoculum inoculation. (**a**) Chlorophyll content, (**b**) TTC reduction strength (the reduction intensity of TTC reflects the vitality of the root system; the higher the value, the stronger the vitality), (**c**) N content, (**d**) P content, (**e**) K content. On the error lines a–e of each graph, it is to judge whether the difference between groups is significant (*p* < 0.05).

The results for the maize inoculated with AMF showed that the plant height and aboveground fresh weight (Figure 8 and Figure 9) of inoculated maize were significantly higher than those of the control, with the plant heights of RG and AG increased by 70% and 80%(Figure 9a,c), respectively, as compared with that of the control, and the aboveground fresh weight of *R.* increased by 146%, compared with that of the control. The fresh weight of the underground part and the dry weight of the above/below ground part inoculated with single-strain AMF (Figure 9c–f) were significantly higher than those of the control, but the inoculation results with the combination strains (*RA*, *RG*, *AG*, *RAG*) were significantly lower than those of the control. There was no significant difference in the above/below water content between the treatments and the control. The sequence of infection rate was as follows (Figure 9h): *A.* > *G.* > *R.* > *AG* > *RA* = *RAG* > *RG*. The root system of *A.* had the strongest infection capacity, and the root infection rate of *RG* was significantly lower than those of other treatment groups. All the treatments improved the maize biomass, and treatment *R.* was the most significant. As shown in Figure 8 and Figure 10, treatment *R.* significantly optimized the maize root architecture: the total root length (+ 50%), root surface area (+ 106%), root projected area (+ 23%), average diameter (+ 157%), number of branches (+ 184%) and root volume (+ 150%) were all significantly higher than those of the control, while the multi-parameters under the mixed bacterial treatment were lower or showed no difference compared with those of the control, indicating that single bacterial inoculation in treatment *R.* had the optimal promoting effect with respect to root development.

The measurement results of chlorophyll and root activity showed the following (Figure 10a,b): The chlorophyll contents in Treatments *R.*, *RA*, *RG*, *AG* and *RAG* were slightly higher than those of the control (the difference between groups was not significant), and those in Treatment *A.* were significantly lower than those of the control. All treatments were higher than the control in root activity, among which *RA* (+1112%), *AG* (+722%) and *RAG* (+1250%) had significant differences from the control, while the other treatments were higher than the control, but the differences were small. The results showed that compound inoculation could significantly improve the root activity of maize seedlings. After inoculation with AMF, the K content in the aerial parts of maize seedlings was higher than that of the control, and the effect of *RG* was the most significant (+4042%), followed by *AG* (+839%). N content in treatment *G.* was significantly higher than CK (+28%), and the differences among the other treatments were small. As for P content, *R.* and *A.* increased by 30% and 57% compared to the control, and were lower in the remaining treatments. In summary, the uptake of K by plants inoculated with AMF was significantly increased, especially under the treatment of *RG*, but the uptake of N and P by plants inoculated with the seven treatments was weakly promoted, or even lower than that of the control.

Further principal component analysis was performed on the data of each index processed by different methods; three principal components were obtained in total, and the comprehensive scores were ranked as shown in Table S6: *R.* > *AG* > *A.* > *RG* > *RAG* > *G* > *RA* > CK. Treatment 1 had the highest score (1.53) and the lowest CK (−0.67). This indicated that single inoculation of *R.* had the most significant growth-promoting effect on maize seedlings.

## 4. Discussion

The results from high-throughput sequencing showed that AMF community in maize rhizosphere in northern Xinjiang covered eleven genera, seven families and three orders, among which Glomerales was the absolute dominant group and *Glomus* was the horizontal dominant group, which was consistent with the results of morphological identification, and confirmed that *Glomus melanosporum* was the dominant species in the arid saline–alkali farmland ecosystem [71]. As Ingrid Lenoir et al. [72] pointed out, this genus has dominated the AMF community in different environments. *Glomus* was able to colonize efficiently with mycelia or mycorrhizal fragments and showed strong adaptability and sporulation [73,74]. Its rapid hyphal growth and high infection efficiency helped to enhance plant nutrient absorption and enhance host competitiveness, thereby further promoting the reproduction and amplification of the genus and maintaining its broad and stable distribution in diverse habitats [74,75]. Random forest analysis further confirmed that the AMF community differences between different places is mainly reflected in *Glomus*, which confirmed the remarkable characteristics of *Glomus’* extensive ecological adaptability to various environments [34]. However, the rhizosphere soil environment more suitable for the growth and reproduction of *Glomus* may have adverse effects on the growth of other AMF genera, thus reducing the overall diversity and abundance of AMF [76]. In particular, there are some technical limitations in this study: a single SSU rDNA marker cannot accurately distinguish the genus-level groups in *Glomus*, and the incompleteness of the database reference sequence makes it difficult to annotate some groups [77]. In the future, this marker and a 28S rDNA multi-gene joint marker strategy can be used, combined with a more comprehensive AMF reference sequence database to further improve the accuracy of species identification, reduce the proportion of unclassified groups and provide a more accurate taxonomic basis for the study of AMF community structure and function [78].

The present study also revealed that the Alpha diversity index of the AMF community in the rhizosphere soil of the T2 sample plot was significantly higher than those in other sample plots. It has been emphasized that the diversity of AMF communities is closely related to the physicochemical properties of soil (such as organic matter content and levels of N, P and K) [79]. Higher total potassium (TK) and lower available phosphorus (AP) and total phosphorus (TP) contents in the T2 sample plot might be more conducive to the colonization and the maintenance of the diversity of AMF. At the same time, the soil S_ACP activity in T2 plot is at a high level, which may reflect soil microbial processes that are more active, including AMF’s own metabolic activities, and thus may support higher microbial diversity. At the same time, the Beta diversity analysis indicated that the direct contribution of sample plot classification to community variation was limited, while environmental factors such as soil nutrients might have a greater impact. On the contrary, the low organic matter in the T1 and S1 plots limited the carbon source supply of AMF, while the high EC value in S1 plots inhibited the mycelium growth and spore germination of AMF through osmotic stress [80]. The double pressure made it difficult for most AMF groups to survive, and the diversity decreased significantly.

In terrestrial ecosystems, soil factors can prove influential. Here soil type, pH and fertility all have an impact on the composition and distribution of AMF communities [81,82]. In summary, TN, S_ACP and TK were the three core environmental factors affecting the AMF community in the present study. TN and S_ACP mainly regulated the overall diversity level of the community, and TK mainly regulated the composition and abundance of specific genera in the community, further confirming that different soil factors played different key roles in shaping the structure and diversity of AMF communities [83,84,85]. S_ACP played an important role in the composition and distribution of AMF communities, while AMF could also affect the availability of soil phosphorus and the structure of microbial communities by regulating acid phosphatase activity [86]. N addition directly led to the decline of AMF species richness by reducing soil pH and increasing nitrate content, and this effect was exacerbated with the treatment time [87]. In contrast, long-term manure application helped to maintain high AMF diversity by increasing soil TN levels [88]. The results showed that nitrogen source was essential for the growth and function of AMF, and different forms (such as TN and AN) had different effects on the composition of the AMF community. Potassium is a key nutrient element for plant growth and participates in water balance, enzyme activity and energy transport, as well as affecting the growth and reproduction of AMF. An appropriate potassium supply indirectly enhanced AMF activity by promoting host plant growth [89]. Yang Shuanglin’s research and the present results show that the content of TK in soil is positively correlated with the relative abundance of *Scutellospora*; this shows that a high-potassium environment promotes the colonization of *Scutellospora*. However, excessive potassium supply may inhibit AMF. Adenan et al. [90,91] found a negative correlation between AK and AMF diversity, which is consistent with the significant negative correlation between *Diversispora* and AK determined in this study. The potential mechanism might be that the high-potassium environment reduced the dependence of plant roots on AMF mineral nutrition (especially potassium) absorption, which in turn might reduce carbon distribution from plants to AMF, inhibit AMF growth and reproduction and reduce community diversity and abundance.

The results of root staining showed that AMF showed significant infection differences in different ways; hyphal infection was the main manifestation of AMF infection in maize roots, indicating that AMF had formed a good symbiotic relationship with maize. Conversely, hyphal infection also determined the total infection rate to a certain extent [92]. According to the morphological identification results, the dominant species was *Glomus melanosporum* (based on the combined HTS amount and morphological identification results), which was consistent with the amount in HTS. The genus *Glomus* was also the dominant genus in all the plots. *Glomus* is widely distributed in a variety of agricultural soils and has demonstrated significant ecological advantages [93]. At the same time, it was found that some genera only appeared in the HTS abundance, which might be due to the fact that the traditional morphological identification methods mainly depended on observing the morphological characteristics of spores, and all species could not be detected, especially those that did not form obvious spores or those that were represented by a small number in the samples, which indicated a certain subjective error [94]. In addition, AMF species have different responses to changes in external environment (such as soil properties, temperature, precipitation, etc.). Therefore, under different environmental conditions, the number of spores of some AMF species may decrease, so some AMF species are not isolated from the nature of the collected soil samples [95,96]. In experimental observations, some AMF species showed high isolation frequency and importance values, probably because their own biological characteristics were suitable for the habitat, and this adaptability was the result of the co-evolution and selection of geographical environment factors and host plants.

In this study, we found that seven treatments (*R.*, *A.*, *G.*, *RA*, *RG*, *AG* and *RAG*) had different effects on maize seedling inoculation, and the comprehensive growth promotion effect of treatment *R.* was the best; in particular, the findings indicated that the species composition of AMF microbial inoculum had different promotion effects on host plants. Such differences may be due to differences in AMF’s ability to infect specific hosts, the efficiency of the mineral element transfer to hosts, carbon demand, and compatibility with host plants [97]. However, for host plants inoculated with different varieties of the same AMF, there were also differences in root system, root-to-shoot ratio and specific root length. The results showed that inoculation with AMF significantly increased the overall growth and yield of maize. In the study of Rashwan et al. [98], inoculation of AMF resulted in the highest yield-related traits for wheat in both seasons. Jia et al. found that AMF inoculation could inhibit root elongation of intercropped soybean [99], while *Glomus mosseae* inoculation could significantly increase soybean biomass. The research conducted by Hu Xueyi et al. revealed that AMF had a positive effect on the growth and development of Flaveria bidentis [92]; that is, it significantly promoted the biomass growth in the aboveground part and root of Flaveria bidentis, and these research results were similar to the experimental results in the present paper. It has been revealed that AMF can form a hyphal network with plant roots, enhance the access of roots to soil surface area, improve plant nutrition by increasing the availability and transport of various nutrients and affect soil structure and texture to improve soil quality and thus improve plant growth [100]. Multiple studies have suggested that inoculation of AMF can promote the absorption of N, P and other elements in plants [101]. In this study, after inoculation with AMF, it was found that the plant’s K absorption was significantly increased, especially under the treatment of RG, but the promotion of N and P absorption by the plants inoculated with the seven treatments was weak, or even lower than that of the control. During the planting process, the phosphorus concentration in the applied nutrient solution was high, while the ability of AMF to assist the host plant in phosphorus absorption was decreased when the phosphorus concentration was too high. In general, AMF helps plants expand their absorption range and improve transport efficiency only when the concentrations of directly available inorganic phosphorus in the soil are low and the absorption needs of the plants are not being met. For example, AMF-inoculated okra was automatically regulated in response to adverse stress under low phosphorus and drought conditions [102]. However, whether inoculation with AMF can improve nitrogen uptake by plants is still controversial. The physiological and biochemical mechanisms of this process have not been clarified. The experimental results may be affected by the species inoculated with AMF, soil nutrition, host plants and ecosystem selection. The impacts of AMF on host plants are manifold, including changing plant succession and distribution, promoting nutrient absorption, promoting development and growth, enhancing stress resistance, and improving the quality and yield of target products. As a beneficial soil microorganism, AMF provides a variety of ecological functions, enhancing soil fertility and promoting plant growth [103].

In addition, we found that the growth-promoting effect of AMF compound was significantly lower than that of the single-bacterium treatment. For example, the fresh weight, dry weight and a large number of elements relating to the P of maize under the compound combination were lower than those under the single-bacterium treatment group. There are many mechanisms behind this phenomenon. First of all, different AMF strains have significant differences in nutrient absorption efficiency, mycelium growth rate and space occupation ability, causing resource competition [104,105]. This competition directly leads to the decline in the biomass of some strains and reduces the overall efficiency of the compound system [105]. Secondly, some AMF strains (such as *Glomus mosseae*) can secrete antibiotic-like substances, which can specifically interfere with the expression of symbiotic related genes (such as RAM1 and RAM2) and block the process of hyphae invading the host root system, thus inhibiting the colonization of neighboring strains [106,107]. Thirdly, AMF single-bacterium can specifically utilize the specific nutrient resources around the root system of host plants. For example, under phosphorus (P) stress, in the co-infection experiment involving *Glomus* sp. and *S. calospora* on clover roots, *S. calospora’s* inhibitory effect on *Glomus* sp. was significantly stronger than the reverse effect. This competition stems from the difference in the utilization efficiency of different AMF species: when the soil P level increases, the colonization rate of *Glomus* sp. drops sharply, while that of *S. calospora* remains stable, indicating that the competition outcome is directly regulated by the availability of resources [108]. In addition, AMF regulates nutrient flow through the mycelium network. For example, the distribution direction of nitrogen is influenced by the composition of AMF community, thus changing the interspecific competition patterns of plants [109]. The interaction mechanism between different AMF strains can be further explored in future research, developing the combinations of strains with synergistic effects to improve the application effect of AMF in agricultural production.

## 5. Conclusions

In this study, the community characteristics and functional values of AMF in maize rhizosphere in a saline–alkali area of northern Xinjiang were analyzed by high-throughput sequencing, morphological identification and a pot experiment. The results showed that the AMF community in this area covered three orders, seven families and eleven genera; *Glomus* was the absolute dominant group, and the T2 plot in Yumin County had the highest species diversity. Soil TN, S_ACP and TP are the core factors regulating AMF community structure. The single inoculation of the dominant strain *Rhizophagus intraradices* was significantly better than compound inoculation in promoting the growth of maize; this strain could be used as a priority candidate strain for the research and development of maize microbial inoculum in saline–alkali soil. The study provided a theoretical basis for the utilization of AMF resources in saline–alkali soil.

## Figures and Tables

**Table 1:** AMF species classification of maize rhizosphere soil in Xinjiang.

Phylum Glomeromycota Class Glomeromycetes Orders (3)	Families (7)	Genera (11)
Glomerales	Glomeraceae	*Glomus*
		*Septoglomus*
		*Rhizophagus*
		unclassified_*Glomeraceae*
	Claroideoglomeraceae	*Claroideoglomus*
	unclassified_Glomerales	unclassified_*Glomerales*
Diversisporales	Acaulosporaceae	*Acaulospora*
	Diversisporaceae	*Diversispora*
	Gigasporaceae	*Scutellospora*
		*Gigaspora*
unclassified_Glomeromycetes	unclassified_Glomeromycetes	unclassified_*Glomeromycetes*

**Table 2:** Morphological classification system of AMF species in maize rhizosphere soil in Xinjiang.

Phylum: Glomeromycota Class: Glomeromycetes Orders (3)	Families (6)	Genera (9)	Species (14)
Glomerales	Glomeraceae	*Glomus*	*Glomus melanosporum*
			*Glomus clarum*
			*Glomus constrictum*
		*Rhizophagus*	*Rhizophagus intraradices*
		*Septoglomus*	*Septoglomus constrictum*
Diversisporales	Acaulosporaceae	*Acaulospora*	*Acaulospora denticulata*
			*Acaulospora elegans*
			*Acaulospora tuberculata*
		*Entrophospora*	*Entrophospora colombiana*
	Diversisporaceae	*Diversispora*	*Diversispora spuecum*
	Gigasporaceae	*Scutellospora*	*Scutellospora calospora*
Archaeosporales	Archaeosporaceae	*Archaeospora*	*Archaeospora leptoticha*
			*Archaeospora schenckii*
	Ambisporaceae	*Ambispora*	*Ambispora jimgerdemannii*

**Table 3:** Inter-root AMF distribution in maize and its IF, RA and IV.

Species	IF	RA	IV	Region
*Glomus melanosporum*	95.00%	38.33%	66.66%	M1–M3, H1, H2, S1, S2, S3, B1–B3, T1–T4, Y1–Y4
*Glomus clarum*	20.00%	5.56%	12.78%	H3, B3, Y3, Y4
*Glomus constrictum*	50.00%	8.72%	29.36%	M2, M3, H1, S1, B2, T2, T3, Y2, Y4
*Rhizophagus intraradices*	85.00%	30.25%	56.46%	M1–M3, H1–H3, S1, S2, S3, B1–B3, T1–T4
*Septoglomus constrictum*	60.00%	7.03%	33.51%	M1–M3, H1, H2, S1, S2, B1, B3, T1, T4, Y3
*Acaulospora denticulata*	60.00%	8.85%	34.43%	M1, M3, H3, S1–S3, B1, B3, T1, T4, Y1, Y3
*Acaulospora elegans*	10.00%	1.06%	5.53%	M2, S2
*Acaulospora tuberculata*	10.00%	0.49%	5.24%	M2, S2
*Entrophospora colombiana*	15.00%	0.68%	7.84%	M1, M2, S1
*Diversispora spuecum*	50.00%	7.38%	28.69%	H1, H2, B2, B3, T2, T3, T4, Y2, Y3, Y4
*Scutellospora calospora*	20.00%	3.55%	11.78%	H2, B3, T4, Y3
*Archaeospora leptoticha*	75.00%	10.07%	42.54%	M3, H1, H3, S2, S3, B1, B2, T1–T4, Y1, Y2, Y4
*Archaeospora schenckii*	60.00%	4.91%	32.46%	M3, H1, H3, S2, S3, B1, B2, T1, T2, Y2, Y4
*Ambispora jimgerdemannii*	10.00%	3.38%	6.69%	H2, T4

Note: The IF column represents the separation frequency, and the frequency or probability of successfully separating AMF from maize rhizosphere. The column RA indicates relative abundance, which reflects the difference of richness of each species in the community. Column IV reports the important value, which combines the abundance and frequency of species, and the important values of different species are obviously different.

**Table 4:** Colonization of AMF in maize rhizosphere.

Region	Sample Plot Number	Vesicles	Arbuscules	Hyphae	Overall Infestation Rate	Average Total Infection Rate in Each Region
Changji Hui autonomous prefecture	M1	4.68%	8.54%	34.27%	62.46%	72.24%
	M2	2.15%	16.49%	40.14%	83.51%	
	M3	3.13%	13.48%	35.74%	66.02%	
	H1	17.98%	6.27%	38.15%	74.66%	
	H2	0.31%	16.21%	45.26%	76.76%	
	H3	6.60%	12.18%	35.03%	70.05%	
ShiHeZi	S1	2.61%	7.35%	38.15%	79.38%	68.60%
	S2	3.53%	13.04%	30.98%	56.79%	
	S3	1.47%	13.27%	44.25%	69.62%	
Bortal Mongolian Autonomous Prefecture	B1	37.18%	3.85%	15.38%	57.05%	64.79%
	B2	2.94%	34.12%	17.06%	60.59%	
	B3	3.82%	19.08%	36.26%	76.72%	
Ilikazak Autonomous Prefecture	Y1	1.31%	10.76%	47.51%	72.97%	72.41%
	Y2	0.93%	12.77%	41.12%	71.03%	
	Y3	7.75%	9.25%	37.75%	76.75%	
	Y4	5.20%	7.43%	41.83%	75.50%	
	T1	0.97%	11.97%	37.86%	67.64%	
	T2	6.35%	9.79%	41.80%	78.31%	
	T3	2.86%	9.35%	38.70%	69.09%	
	T4	5.08%	9.64%	21.83%	68.02%	

**Table 5:** Pearson correlation analysis for soil factors and root infestation rates.

	Pearson Correlation	Sig. (Double Tail)
pH	0.113	0.634
EC	−0.129	0.589
SOM	−0.116	0.627
TN	−0.039	0.872
TP	0.062	0.795
TK	0.233	0.324
AN	0.029	0.905
AP	0.035	0.883
AK	−0.253	0.282
S_ACP	0.155	0.513
PROT	−0.034	0.886
SAC	−0.056	0.814

## Data Availability

The HTS data of this study has been uploaded to NCBI (SRA), BioProject: PRJNA1365752. The sequence data of three AMF strains involved in this study have been uploaded to NCBI. *Rhizophagus intraradices*, *Acaulospora denticulata* and *Glomus melanosporum* obtained NCBI accession numbers PX627800, PX627801 and PX627802, respectively.

## Supplementary Materials

The following supporting information can be downloaded at: https://www.mdpi.com/article/10.3390/jof12010027/s1, Table S1: Geographic information table of sample collection points in northern Xinjiang; Table S2: Ecological parameters of structural diversity of AMF communities; Figure S1: Dilution curve of maize rhizosphere soil in northern Xinjiang; Table S3. Classification system of AMF species in inter-root soil; Table S4. Physical and chemical properties of soils in different locations in northern Xinjiang; Table S5. Soil enzyme activities in maize rhizosphere at different locations in northern Xinjiang; Figure S2: Phylogenetic tree of AMF constructed based on the neighbor-joining method (NJ method). *A.* (*Acaulospora denticulata*); *G.* (*Glomus melanosporum*); *R.* (*Rhizophagus intraradices*); Table S6. Principal component analysis for each treatment.

## Abbreviations

The following abbreviations are used in this manuscript:

AMF	Arbuscular Mycorrhizal Fungi
ASVs	Amplicon sequence variants
RDA	Redundancy Analysis
TK	Total Potassium
TN	Total Nitrogen
TP	Total Phosphorus
AK	Available Potassium
AN	Available Nitrogen
AP	Available Phosphorus
SOM	Soil Organic Matter
EC	Electrical Conductivity
PRO	Protein
SUR	Sucrase
S_ACP	Soil Acid Phosphatase
R.	*Rhizophagus intraradices*
A.	*Acaulospora denticulata*
G.	*Glomus melanosporum*
RA	*Rhizophagus intraradices * Acaulospora denticulata*
RG	*Rhizophagus intraradices * Glomus melanosporum*
AG	*Acaulospora denticulata * Glomus melanosporum*
HTS	High-throughput sequencing
