# Supplementary material for: Diversity of Arbuscular Mycorrhizal Fungi in Rhizosphere Soil of Maize in Northern Xinjiang, China, and Evaluation of Inoculation Benefits of Three Strains
Source: J Fungi (Basel). 2025 Dec 29;12(1):27. doi: 10.3390/jof12010027 (PMC12843313; doi:10.3390/jof12010027)
Supplement: Supplementary file 1 [file jof-12-00027-s001.zip › jof-4054913-supplementary.pdf]

Table S1. Geographic information table of sample collection points in northern Xinjiang.

Studied sites	area	location	Sample codes	Longitude (E)	Latitude (N)
1	Changji Hui Autonomous Prefecture	Manasi	M1	86.18°	44.33°
2		Manasi	M2	86.30°	44.26°
3		Manasi	M3	86.33°	44.25°
4		Hutubi	H1	86.71°	44.19°
5		Hutubi	H2	86.86°	44.14°
6		Hutubi	H3	86.98°	44.06°
7	Shihezi	Shihezi	S1	86.10°	44.27°
8		Shihezi	S2	85.87°	44.25°
9		Shihezi	S3	85.79°	44.25°
10	Bortal Mongolian Autonomous Prefecture	Shuanghe	B1	82.55°	44.87°
11		Bole	B2	81.81°	44.97°
12		Wenquan	B3	81.56°	45.04°
13	Yili Kazak Autonomous Prefecture	Huocheng	Y1	80.85°	44.25°
14		Huoerguosi	Y2	80.66°	44.18°
15		Kedala	Y3	80.52°	44.06°
16		Nileke	Y4	82.56°	43.80°
17		Tuoli	T1	83.61°	46.03°
18		Yumin	T2	83.24°	46.29°
19		Tacheng	T3	83.07°	46.67°
20		Tacheng	T4	82.95°	46.79°

Table S2. Ecological parameters of structural diversity of AMF communities.

ecological parameters	Calculation formula / standard
Spore density, SD	Refers to the total number of AMF spores contained in each 50g air-dried soil sample.
Species richness, SR	Refers to the number of species in a habitat. In this study, it refers to the number of spore species in 50 g soil.
Isolation frequency, IF	IF = (AMF the number of soil samples that appear. / total number of soil samples) × 100%
Relative abundance, RA	RA = (AMF spore number of a genus or species / AMF total spore number) × 100%
Importance value, IV	I = (F + RA) / 2

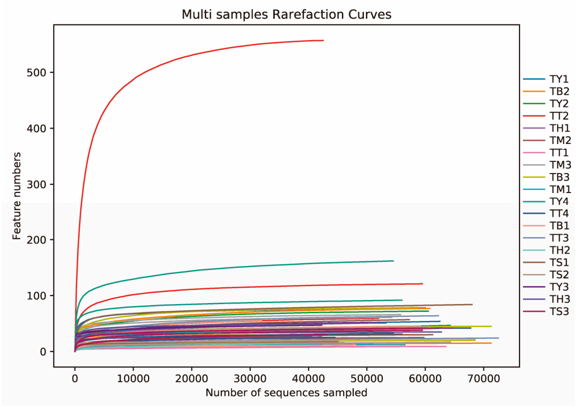

Figure S1. Dilution curve of maize rhizosphere soil in northern Xinjiang.

Table S3. Classification system of AMF species in inter-root soil.

ample	ACE	Chao1	Simpson	Shannon
M1	55.78 ± 22.73b	20.50±7.76b	0.74±0.11ab	2.48±0.48cd
M2	46.26 ± 24.79b	31.17±10.86b	0.82±0.05ab	3.06±0.54bcd
M3	61.97 ± 21.23b	42.46±13.74b	0.74±0.16ab	3.15±0.92bcd
H1	27.67 ± 12.34b	27.00±12.01b	0.77±0.10ab	2.90±0.80bcd
H2	30.44±10.99b	23.25±8.27b	0.76±0.07ab	2.42±0.48cd
H3	49.83±14.76b	37.67±6.57b	0.87±0.03a	3.59±0.34abcd
S1	21.58±7.85b	46.67±24.46b	0.67±0.05ab	2.50±0.43cd
S2	32.71±12.13b	25.83±7.92b	0.67±0.17ab	2.49±0.76cd
S3	46.10±11.28b	36.75±2.54b	0.73±0.04ab	2.49±0.11cd
B1	44.42±20.06b	42.33±14.77b	0.76±0.13ab	2.95±0.91bcd
B2	27.04±8.61b	49.33±28.26b	0.66±0.21ab	2.77±1.07cd
B3	37.45±2.39b	48.32±18.04b	0.84±0.06ab	3.27±0.55abcd
T1	13.91±4.41b	12.67±4.18b	0.49±0.15b	1.43±0.49d
T2	249.84±152.76a	242.13±157.04a	0.95±0.02a	5.47±1.04a
T3	39.08±18.72b	37.53±18.93b	0.73±0.08ab	2.47±0.64cd
T4	35.22±5.91b	33.33±5.78b	0.77±0.04ab	2.73±0.28cd
Y1	54.21±13.96b	52.33±14.01b	0.82±0.13ab	3.72±1.03abcd
Y2	43.97±20.44b	42.40±19.33b	0.78±0.13ab	3.24±1.02abcd
Y3	117.33±70.04b	52.33±8.11b	0.96±0.00a	4.77±0.13abc
Y4	92.71±37.47b	89.57±35.37b	0.96±0.01a	5.13±0.46ab

Note: Different lowercase letters after the same column of data indicate significant differences ($p < 0.05$).

Table S4. Physical and chemical properties of soils in different locations in northern Xinjiang.

sample	pH	EC (μS/cm)	SOM (g/kg)	AN (mg/kg)	AP (mg/kg)	AK (mg/kg)	TN (g/kg)	TP (g/kg)	TK(g/kg)
**M1**	7.19±0.13gh	361.67±0.88a	14.93±2.78ef	107.60±0.31e	26.99±0.33b	266.00±5.77e	1.19±0.00efg	7.93±0.32b	14.54±0.03e
**M2**	7.05±0.09hi	256.00±1.15e	23.41±0.83cd	76.20±0.31h	10.44±0.19ef	324.00±2.31c	1.37±0.01cde	7.04±0.23cd	15.33±0.00c
**M3**	7.32±0.01efg	262.33±1.45c	11.60±0.75fg	205.23±0.28b	9.97±0.15f	155.00±1.73h	1.68±0.01b	4.77±0.24jkl	15.00±0.10d
**H1**	7.19±0.09gh	286.00±1.73b	38.32±0.40a	65.80±0.00j	22.97±0.19c	341.00±1.73b	1.05±0.01gh	6.92±0.22cde	14.71±0.15e
**H2**	7.21±0.01g	255.00±2.65e	13.09±3.03fg	65.80±0.00j	11.36±0.19ef	180.00±1.15g	1.09±0.03fg	6.08±0.02efgh	14.20±0.01f
**H3**	7.28±0.03fg	257.00±1.53de	30.18±5.74b	59.33±0.44il	10.44±0.26ef	205.00±9.81f	1.00±0.02ghi	5.85±0.01fghi	15.92±0.05b
**S1**	7.41±0.02def	211.67±0.88h	4.15±0.83h	55.57±0.34m	10.13±0.05ef	145.00±1.73i	0.79±0.01ij	5.15±0.35hijk	13.34±0.00h
**S2**	7.44±0.04cde	213.67±1.86h	28.91±1.65bc	65.80±0.00j	9.09±0.10f	282.00±1.15d	1.01±0.02ghi	5.80±0.16fghi	13.59±0.07g
**S3**	7.48±0.01bcd	175.73±1.79k	13.31±1.39fg	42.50±0.29n	9.64±0.13f	202.00±0.00f	0.57±0.02j	5.62±0.11ghij	15.10±0.11d
**T1**	7.63±0.04ab	121.73±0.70m	13.78±0.23fg	68.95±0.00i	5.14±0.22gh	209.00±4.04f	0.70±0.02j	5.90±0.02fghi	14.02±0.03f
**T2**	7.46±0.04cde	261.67±0.88cd	7.25±3.68gh	56.12±0.17m	3.52±0.19h	153.00±2.89hi	0.70±0.01j	3.93±0.09lm	19.58±0.05a
**T3**	7.52±0.02bcd	248.00±1.53f	39.46±0.23a	142.30±0.26c	25.84±0.15b	158.00±0.00h	2.53±0.00a	5.08±0.48ijk	12.16±0.08j
**T4**	7.49±0.01bcd	191.90±0.78i	15.78±1.21ef	76.40±0.26h	6.48±0.33g	188.00±0.00g	0.79±0.35hij	4.43±0.01klm	12.67±0.04i
**B1**	7.01±0.07i	246.67±1.20f	16.16±0.82ef	62.73±0.22k	22.98±3.26c	351.00±4.04a	0.98±0.00ghi	4.99±0.07ijk	11.88±0.01k
**B2**	6.98±0.06ij	228.67±0.88g	11.95±1.50fg	86.30±0.26g	12.53±0.19e	185.00±1.73g	1.46±0.00bcd	6.68±0.30def	12.25±0.04j
**B3**	6.84±0.00j	140.03±1.70l	7.58±2.27gh	111.40±0.26d	54.94±0.54a	183.00±2.89g	1.47±0.00bcd	9.97±0.55a	13.40±0.07gh
**Y1**	7.47±0.03bcde	184.67±2.60j	11.24±1.25fg	65.80±0.00j	19.96±0.14d	183.00±2.89g	1.65±0.02b	5.64±0.13cgij	10.57±0.10m
**Y2**	7.49±0.02bcd	261.67±2.19cd	20.89±0.46cde	76.60±0.21h	21.24±0.18cd	278.00±1.15d	1.57±0.02bc	7.62±0.10b	11.08±0.10l
**Y3**	7.76±0.01a	140.90±1.32l	12.40±2.82fg	250.73±0.38a	10.99±0.13ef	99.00±2.89j	0.98±0.00ghi	3.82±0.08m	11.70±0.19k
**Y4**	7.58±0.03bc	257.67±1.86cde	29.60±0.83cb	88.70±0.38f	22.83±0.21c	268.00±1.15e	1.30±0.01def	6.39±0.79defg	11.78±0.01k

Note: Different lowercase letters after the same column of data indicate significant differences ($p < 0.05$).

Table S5. Soil enzyme activities in maize rhizosphere at different locations in northern Xinjiang.

sample	PRO (mg/g)	SUR (mg/g)	S_ACP (U/g)	sample	PRO (mg/g)	SUR (mg/g)	S_ACP (U/g)
**M1**	3.46±0.10gh	8.69±0.12cd	11744.55±0.00bcde	**T2**	7.96±0.68def	7.06±0.02e	10577.64±572.08cde
**M2**	4.18±0.21g	8.72±0.08cd	10886.70±822.50bcde	**T3**	7.19±0.61ef	9.76±0.35ab	11406.14±3230.39bcde
**M3**	6.13±0.27f	9.26±0.06abc	14686.85±2215.01abc	**T4**	1.98±0.53hi	9.26±0.15abc	12891.19±334.79bcde
**H1**	1.23±0.25i	8.69±0.08cd	10080.95±0.00cde	**B1**	8.2±0.55de	8.32±0.02cd	9325.67±472.57e
**H2**	1.16±0.20i	9.89±0.06a	17543.08±989.72a	**B2**	8.27±0.54de	8.95±0.10bcd	11345.74±909.10bcde
**H3**	2.39±0.48hi	8.49±0.09cd	14171.20±1120.01abcd	**B3**	19.16±1.15a	9.98±0.50a	15285.14±1671.60ab
**S1**	9.78±0.50cd	8.09±0.16d	11981.79±0.00bcde	**Y1**	6.73±0.72ef	9.99±0.32a	9992.74±1969.13de
**S2**	12.84±0.45b	8.68±0.05cd	11792.76±392.11bcde	**Y2**	9.44±0.64cd	9.23±0.08abc	11600.18±3017.55bcde
**S3**	8.35±0.95de	7.00±0.07e	11256.40±158.51bcde	**Y3**	6.95±0.02ef	6.51±0.15e	10733.14±0.00 bcde
**T1**	1.91±0.50hi	6.86±0.05e	9867.31±960.50de	**Y4**	10.21±0.80c	8.64±0.99cd	11347.36±106.00bcde

Note: Different lowercase letters after the same column of data indicate significant differences ($p < 0.05$).

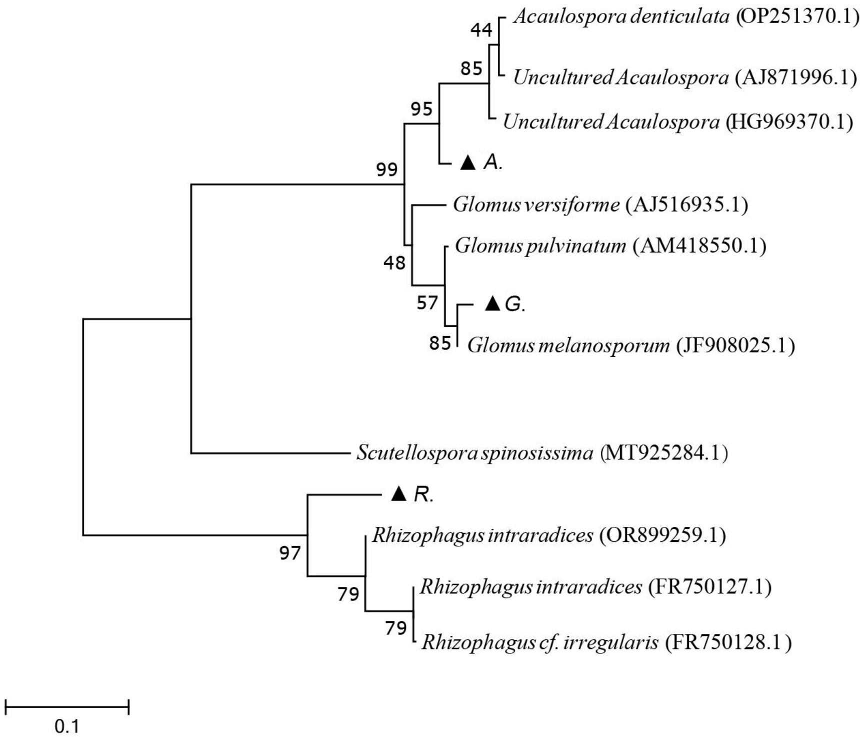

Figure S2. Phylogenetic tree of AMF constructed based on the neighbour-joining method (NJ method). *A.* (*Acaulospora denticulata*); *G.* (*Glomus melanosporum*); *R.*(*Rhizophagus intraradices*).

Table S6. Principal component analysis for each treatment.

Treatment	Primary principal component		Secondary principa l component		Tertiary Principal Component		Composite score	Ranking In composite score
	Score	Rank	Score	Rank	Score	Rank		
CK	-0.51336	5	-1.35334	8	0.77029	2	-0.67	8
R.	2.09976	1	0.77644	3	0.27182	5	1.53	1
A.	0.87976	2	-1.00265	6	-0.85719	7	0.15	3
G.	-0.06374	3	-1.18609	7	0.32306	4	-0.39	6
RA	-0.64613	7	0.49164	4	-2.07942	8	-0.4	7
RG	-0.58899	6	0.88108	2	0.33764	3	-0.05	4
AG	-0.35589	4	0.98402	1	1.00064	1	0.18	2
RAG	-0.8114	8	0.40891	5	0.23316	6	-0.34	5
